# The Dynamics of *Microcystis* Genotypes and Microcystin Production and Associations with Environmental Factors during Blooms in Lake Chaohu, China

**DOI:** 10.3390/toxins6123238

**Published:** 2014-12-02

**Authors:** Li Yu, Fanxiang Kong, Min Zhang, Zhen Yang, Xiaoli Shi, Mingyong Du

**Affiliations:** 1 State Key Laboratory of Lake Science and Environment, Nanjing Institute of Geography and Limnology, Chinese Academy of Sciences, Nanjing 210008, China; E-Mails: yuli514605@163.com (L.Y.); mzhang@niglas.ac.cn (M.Z.); zhyang@niglas.ac.cn (Z.Y.); xlshi@niglas.ac.cn (X.S.); dmy19890208@163.com (M.D.); 2 College of Resources and Environment, University of Chinese Academy of Sciences, Beijing 100049, China

**Keywords:** *Microcystis*, microcystin, 16S rDNA, *mcyD*, qPCR, environmental factors, Lake Chaohu

## Abstract

Lake Chaohu, which is a large, shallow, hypertrophic freshwater lake in southeastern China, has been experiencing lake-wide toxic *Microcystis* blooms in recent decades. To illuminate the relationships between microcystin (MC) production, the genotypic composition of the *Microcystis* community and environmental factors, water samples and associated environmental data were collected from June to October 2012 within Lake Chaohu. The *Microcystis* genotypes and MC concentrations were quantified using quantitative real-time PCR (qPCR) and HPLC, respectively. The results showed that the abundances of *Microcystis* genotypes and MC concentrations varied on spatial and temporal scales. *Microcystis* exists as a mixed population of toxic and non-toxic genotypes, and the proportion of toxic *Microcystis* genotypes ranged from 9.43% to 87.98%. Both Pearson correlation and stepwise multiple regressions demonstrated that throughout the entire lake, the abundances of total and toxic *Microcystis* and MC concentrations showed significant positive correlation with the total phosphorus and water temperature, suggesting that increases in temperature together with the phosphorus concentrations may promote more frequent toxic *Microcystis* blooms and higher concentrations of MC. Whereas, dissolved inorganic carbon (DIC) was negatively correlated with the abundances of total and toxic *Microcystis* and MC concentrations, indicating that rising DIC concentrations may suppress toxic *Microcystis* abundance and reduce the MC concentrations in the future. Therefore, our results highlight the fact that future eutrophication and global climate change can affect the dynamics of toxic *Microcystis* blooms and hence change the MC levels in freshwater.

## 1. Introduction

Harmful cyanobacterial blooms pose a threat to freshwater ecosystems used for recreation and drinking water supply because cyanobacteria can synthesise toxic secondary metabolites, such as cyanotoxins [1,2,3]. Microcystins (MCs) are the most widespread cyanotoxins present in freshwater and act as a protein phosphatase inhibitors and tumour promoters, causing acute and chronic poisoning in humans and animals, particularly liver injury [4,5,6,7]. MCs are produced by diverse cyanobacterial genera, including *Microcystis*, *Anabaena*, *Planktothrix*, *Aphanizomenon*, *Nostoc*, and *Phormidium* [8,9]. Among them, *Microcystis* is considered to be the most prominent contributor to the production of MCs [10,11,12].

*Microcystis* populations are usually composed of toxigenic and non-toxigenic strains in the aquatic system [13,14,15]. Different *Microcystis* strains show varying responses to different environmental variables [16,17]. The successive replacement of toxigenic and non-toxigenic strains during the development of cyanobacterial blooms has been suggested to be the cause of the changes in MC levels [18]. However, it is not possible to distinguish between toxigenic and non-toxigenic strains of *Microcystis* using traditional techniques, such as morphological and pigment analyses. Recently, quantitative real-time PCR (qPCR) has been developed and widely used to estimate toxic *Microcystis* genotype abundance in natural populations based on specific MC synthetase genes (*mcy*) [19,20,21,22,23]. Moreover, many studies have demonstrated key factors affecting the abundance of toxic *Microcystis* genotypes and MC concentrations in different freshwater ecosystems. In Lake Erie, Rinta-Kanto *et al.* [24] have reported that the total phosphorus concentration is positively correlated with the *mcyD* genotype and MC concentrations. The relative abundance of the *mcyA* genotype has been shown to increase with high nitrate loading in Lake Mikata, Japan [19], whereas strong positive correlations between water temperature, MC concentrations, and *mcyE* and *mcyB* copy number have been found in the Hartbeespoort and Roodeplaat reservoirs of South Africa [25].

Lake Chaohu, which is located in Auhui Province of southeastern China (31°40'N, 117°36'E), is the fifth largest freshwater lake in China (surface area: 760 km^2^). It is an important fishery and drinking water resource for more than 9.66 million people in Chaohu and Heifei city [26,27,28]. Due to rapid economic development and excessive exploitation of the environment, Lake Chaohu is in a eutrophic state. Since the 1980s, this lake has experienced massive cyanobacterial blooms each year during the warm seasons, with a predominance of *Microcystis* spp. [29,30]. Meanwhile, MC pollution in the lake is becoming more serious, and it is common for its MC concentrations of Lake Chaohu to exceed the provisional guideline of 1 µg L^−1^ set by the WHO [31]. Furthermore, previous observations at Lake Chaohu have shown that seasonal variation in MC concentrations at different sample stations and have demonstrated that the MC concentrations is correlated with water temperature and nitrogen and phosphorus levels [28,31]. Although environmental variables may affect toxicity by an order of magnitude, the overall MC concentrations in a bloom may be determined by the abundance and proportion of toxic genotypes [32,33]. However, few studies have addressed the dynamics of toxic *Microcystis* genotypes in Lake Chaohu and the correlations of toxic genotypes with environmental factors until now.

The purpose of this study was to investigate the relationships between MC production and the genotypic composition of the *Microcystis* community together with the environmental conditions in Lake Chaohu during bloom periods (from June to October in 2012). We applied qPCR to quantify specific target genes for *Microcystis* and toxic *Microcystis* to determine the genotypic composition of the natural *Microcystis* population. MC concentrations were considered together with qPCR data to verify the correlation between MC concentrations and the abundance of *Microcystis* genotypes in the lake. Moreover, the significant environmental factors that strongly influence the variations in the MC concentrations and the *Microcystis* population were identified through stepwise multiple regressions.

## 2. Results

### 2.1. Variations in Environmental Factors

**Figure 1:**
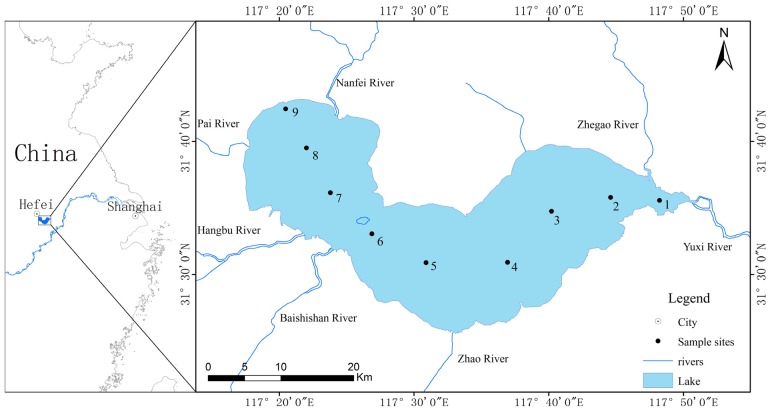
Map of Lake Chaohu with sampling sites.

Physical and chemical parameters from monthly water samples collected at nine sampling sites during June and October 2012 are shown in Table 1 and Table S1 (Supplementary Materials). Sites 1–3 were located in the eastern part of Lake Chaohu, sites 4–6 were located in the centre of the lake, and sites 7–9 were located in the western part of the lake (Figure 1). During the study periods, the lowest water temperature (18.43 °C) was recorded in October, while the highest (30.75 °C) occurred in August. The chlorophyll-a (Chl-a) concentrations varied from 5.70 to 89.40 μg L^−^^1^, and the mean concentration of Chl-a in the western part of the lake was significantly higher than those in the eastern (*p* < 0.01) and central parts (*p* < 0.05). Similarly, nutrient concentrations varied typically among the nine sampling sites. The mean nutrient levels at the western sampling sites were significantly higher than those at the eastern (*p* < 0.01) and central sampling sites (*p* < 0.05). As the bloom developed, nitrate (NO_3_^−^–N), nitrite (NO_2_^−^–N) and ammonium (NH_4_^+^–N) decreased, while total nitrogen (TN), total phosphorus (TP) and orthophosphate (PO_4_^3^^−^–P) concentrations fluctuated. In addition, pH and dissolved oxygen (DO) also varied significantly among the different sampling sites during the bloom season. However, the concentrations of dissolved inorganic carbon (DIC) showed an opposite trend; the highest value was observed in the eastern part of the lake and the lowest in the western part.

**Table 1:** Environmental variables summarised as the mean values and ranges in Lake Chaohu from June to October 2012.

Parameter	Eastern Lake	Central Lake	Western Lake
TN (mg L^−1^)	0.92 (0.56–1.48)	1.44 (0.70–0.25)	2.84 (1.45–5.09)
NH_4_^+^–N (mg L^−1^)	0.15 (0.04–0.42)	0.17 (0.04–0.30)	0.56 (0.09–3.55)
NO_3_^−^–N (mg L^−1^)	0.12 (0.07–0.25)	0.13 (0.06–0.29)	0.36 (0.08–0.70)
NO_2_^−^–N (mg L^−1^)	0.01 (0.00–0.02)	0.01 (0.00–0.06)	0.08 (0.00–0.37)
TP (mg L^−1^)	0.05 (0.03–0.09)	0.11 (0.03–0.23)	0.20 (0.07–0.39)
PO_4_^3−^–P (mg L^−1^)	0.00 (0.00–0.01)	0.02 (0.00–0.05)	0.06 (0.01–0.26)
DIC (mg L^−1^)	14.55 (13.40–16.22)	13.92 (10.59–17.71)	12.60 (8.74–15.06)
DOC (mg L^−1^)	4.64 (3.40–5.67)	4.78 (4.01–5.52)	5.40 (4.26–7.46)
Chl-a (μg L^−1^)	14.42 (5.70–62.28)	30.19 (7.80–65.43)	37.56 (10.72–89.40)
Water temperature (°C)	25.74 (19.18–30.75)	25.30 (18.44–30.41)	24.98 (18.43–30.04)
pH	7.41 (4.73–9.12)	7.76 (5.51–9.36)	7.83 (6.12–9.40)
DO (mg L^−1^)	9.37 (4.47–14.14)	8.64 (4.79–10.35)	8.34 (4.59–14.97)
Secchi depth (m)	52.33 (28.00–85.00)	35.73 (17.00–90.00)	28.54 (15.00–45.00)
Water depth (m)	3.70 (2.30–4.50)	3.81 (1.90–4.50)	3.21 (1.90–4.00)
Conductivity (μS cm^−1^)	315.07 (280.00–370.00)	289.07 (213.00–361.00)	315.10 (223.00–392.00)

### 2.2. Dynamics of Microcystis Genotypes and MC Concentrations in Water Samples

The real-time PCR data showed log-linear relationships for both the 16S rDNA and *mcyD* gene copy number when the genomic DNA from *M. aeruginosa* PCC7806 was used as a template, and the PCR efficiencies of the 16S rDNA and *mcyD* standard curves were 0.99 and 0.96, respectively. The melting curves of the 16S rDNA and *mcyD* real-time PCR products showed peaks at approximately 89.2 and 84.5 °C, respectively, corresponding to the melting temperature of the standard strain *M. aeruginosa* PCC 7806, thereby demonstrating the reliability of the real-time PCR amplification.

**Figure 2:**
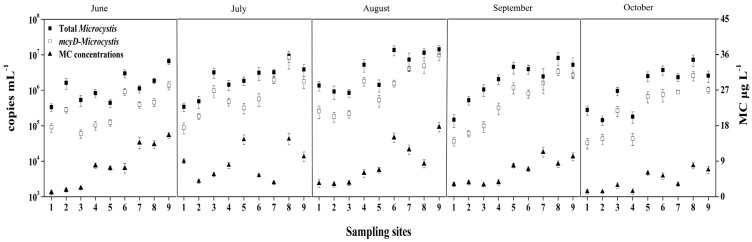
The abundance of total *Microcystis* and toxic *Microcystis* genotypes and MC concentrations at all the water sampling sites in the Lake Chaohu.

**Figure 3:**
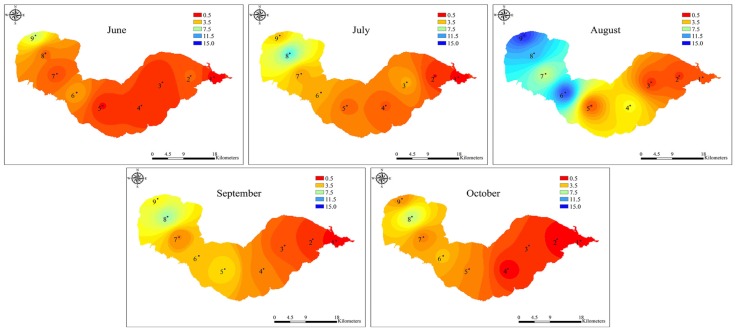
The spatial distribution of total *Microcystis* abundance in Lake Chaohu from June to October 2012. The unit of measurement was 10^6^ copies mL^−^^1^. The interpolation map was constructed by ArcGIS software using the Inverse Distance Weighting method.

**Figure 4:**
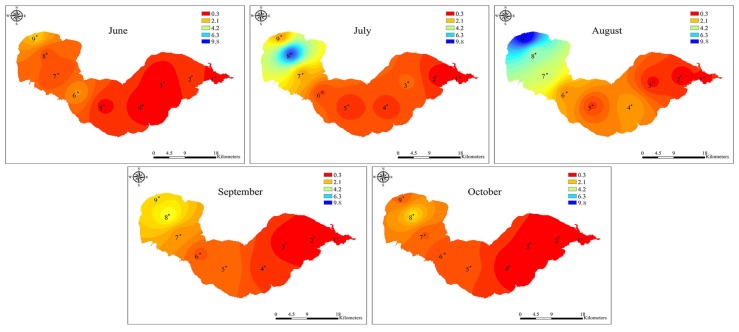
The spatial distribution of toxic *Microcystis* abundance in Lake Chaohu from June to October 2012. The unit of measurement was 10^6^ copies mL^−^^1^. The interpolation map was constructed by ArcGIS software using the Inverse Distance Weighting method.

**Figure 5:**
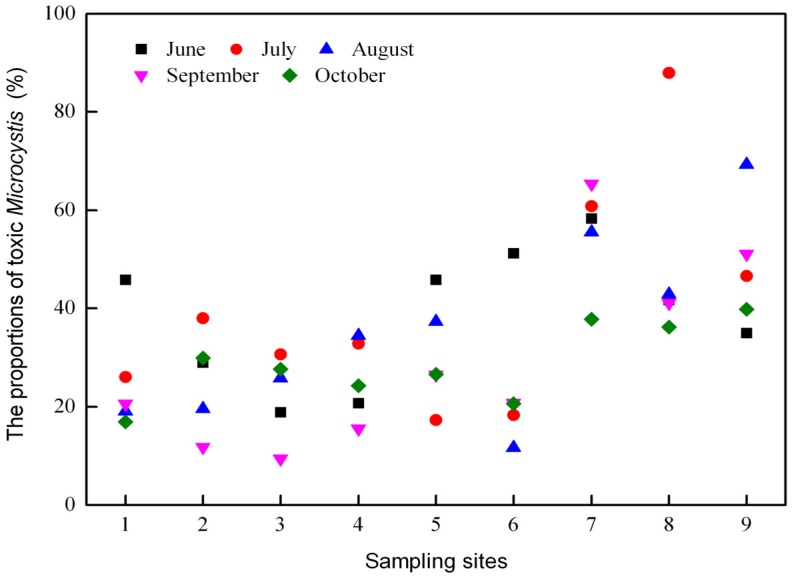
The proportion of toxic *Microcystis* among the total *Microcystis* population in Lake Chaohu from June to October 2012.

Both the total *Microcystis* and potentially toxic *Microcystis* genotypes were detected by real-time PCR in all of the water samples. During the study period, the abundances of total and toxic *Microcystis* genotypes varied from 1.27 × 10^5^ to 1.41 × 10^7^ copies mL^−^^1^ and from 2.63 × 10^4^ to 9.77 × 10^6^ copies mL^−^^1^, respectively (Figure 2). The gene copy numbers for the total and toxic *Microcystis* genotypes at the western sampling sites 7–9 were significantly higher than those at the eastern sampling sites 1–3 (*p* < 0.01) (Figure 3 and Figure 4). The highest copy numbers for the total and toxic *Microcystis* genotypes (1.41 × 10^7^ and 9.77 × 10^6^ copies mL^−^^1^) occurred at site 9 in August, whereas the corresponding lowest values (1.27 × 10^5^ and 2.63 × 10^4^ copies mL^−^^1^) were recorded at sites 1 and 2, respectively, in October. Meanwhile, the proportion of potentially toxic *Microcystis* genotypes within the total *Microcystis* population ranged from 9.43% to 87.98% and showed a trend that was very similar to the abundances of total and toxic *Microcystis* genotypes. The highest proportion was recorded at western sampling site 8 in July, while the lowest value was recorded at eastern sampling site 3 in September (Figure 5).

**Figure 6:**
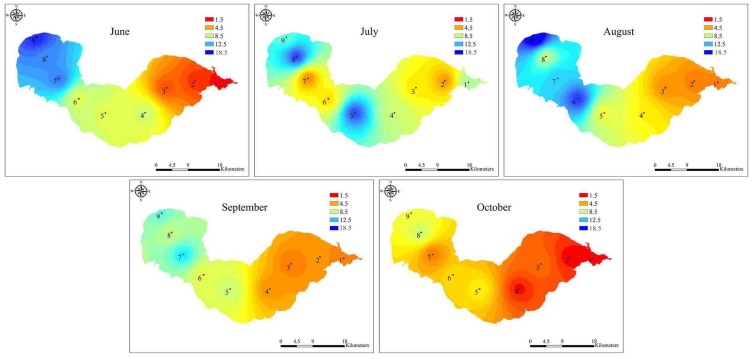
The spatial distribution of total MC concentrations in Lake Chaohu from June to October 2012. The interpolation map was constructed by ArcGIS software using the Inverse Distance Weighting method.

During the bloom period, the total MC concentrations varied across locations and sampling times, ranging from 1.06 to 17.61 μg L^−^^1^, with the highest level occurring at site 9 in August and the lowest detected at site 1 in June (Figure 2 and Figure 6). The MC concentrations in water from Lake Chaohu were above the provisional guideline for drinking water of 1.0 μg L^−^^1^ recommended by the World Health Organization [34].

### 2.3. Relationships of Environmental Variables with MC Concentrations, Microcystis 16S rDNA and mcyD Genotypes Abundance

The Pearson correlation coefficients (*r*) and probability values (*p*) for each correlation are shown in Table 2. The total *Microcystis* 16S rDNA and *mcyD* genotypes were strongly correlated with each other (*r* = 0.941, *p* < 0.001, *n* = 45). The total MC concentrations showed a strong positive correlation with the abundance of *Microcystis* 16S rDNA and *mcyD* copies, and the proportion of toxic genotypes in the *Microcystis* population.

The data showed that the total MC concentrations and the abundance of *Microcystis* 16S rDNA and the *mcyD* copies were positively correlated with TP, TN, NH_4_^+^–N, NO_3_^−^–N, PO_4_^3^^−^–P, water temperature, and pH (*p* < 0.05). Nevertheless, DIC showed a negative relationship with each of the three parameters (*p* < 0.05). In addition, the associations of the Chl-*a* concentration with MC concentrations and the copy numbers of each target gene were positive (*p* < 0.05), but the *mcyD* copy numbers and MC concentrations were negatively correlated with Secchi depth and water depth (*p* < 0.05), and were not correlated with DO or conductivity (*p >* 0.05).

**Table 2:** Pearson correlation coefficients (r) for correlations between environmental factors, *Microcystis* genotypes and microcystins concentration (MC) concentrations (*n* = 45). Significant values (*p* < 0.05) are in bold type.

Variable	*Microcystis* 16S rDNA		Toxic *Microcystis* *mcyD*		MC	
	*r*	*p*	*r*	*p*	*r*	*p*
Toxic *Microcystis* *mcyD*	0.941	**0.000**	-	-	-	-
MC	0.670	**0.000**	0.690	**0.000**	-	-
% toxic proportion	0.310	**0.039**	0.595	**0.000**	0.394	**0.007**
TN	0.532	**0.000**	0.598	**0.000**	0.625	**0.000**
NH_4_^+^–N	0.298	**0.046**	0.351	**0.018**	0.368	**0.013**
NO_3_^−^–N	0.325	**0.029**	0.457	**0.002**	0.289	**0.054**
NO_2_^−^–N	0.273	0.069	0.364	**0.014**	0.381	**0.010**
TP	0.599	**0.000**	0.608	**0.000**	0.675	**0.000**
PO_4_^3−^–P	0.381	**0.010**	0.396	**0.007**	0.379	**0.010**
DIC	−0.467	**0.001**	−0.359	**0.015**	−0.374	**0.011**
DOC	0.395	**0.007**	0.432	**0.003**	0.554	**0.000**
Chl-a	0.562	**0.000**	0.553	**0.000**	0.656	**0.000**
Temperature	0.302	**0.038**	0.334	**0.026**	0.324	**0.022**
pH	0.263	**0.008**	0.314	**0.036**	0.423	**0.004**
DO	−0.051	0.741	−0.038	0.803	0.024	0.875
Secchi depth	−0.365	**0.014**	−0.356	**0.016**	−0.398	**0.007**
Water depth	−0.156	0.308	−0.264	**0.008**	−0.384	**0.009**
Conductivity	−0.126	0.409	−0.002	0.987	−0.006	0.970

### 2.4. Stepwise Multiple Regressions Determining Significant Environmental Variables Correlated with MC Concentrations and Abundance of Microcystis 16S rDNA and mcyD Genotypes (Stepwise Multiple Regression Analyses Were Performed to Determine the Key Environmental Variables that Explained the Abundances of MC and Total and Toxic Microcystis in Lake Chaohu (Table 3))

The first model for predicting total *Microcystis* abundance from environmental variables accounted for over 50% (*R*^2^-adj = 0.53) of the variance and included TP, DIC and water temperature. This model revealed that TP contributed the greatest to the explanation of the variation in the abundance of *Microcystis* 16S rDNA copies (*R*^2^ = 0.36), followed by DIC and water temperature. *Microcystis* 16S rDNA copy abundance was positively correlated with TP and water temperature but negatively correlated with DIC. Thus, the occurrence probability of the *Microcystis* 16S rDNA copies increases with increasing TP and water temperature and decreasing DIC.

A second model relating the abundance of toxic *Microcystis* *mcyD* copies to environmental variables accounted for over 58% (*R*^2^-adj = 0.59) of the variance, with TP, water temperature, DIC and NO_3_^−^-N representing the significant contributors (*p* < 0.05; Table 3). TP also was the most explanatory factor, and each variable had a positive relationship with toxic *Microcystis*, except for DIC. 

A third model describing the MC concentrations accounted for over 70% of the variation (*R*^2^-adj = 0.72). This final model included the following variables: TP, water temperature, and DIC. TP and water temperature were the significant components. Although DIC explained less than TP and water temperature, it was an important explanatory variable for MC concentrations variation. DIC was inversely related to MC concentrations, such that the MC concentrations increased as DIC decreased.

**Table 3:** Three multiple linear regression models for predicting total and toxic *Microcystis* abundance and total MC concentrations in Lake Chaohu. The corresponding *p*-values and coefficients are given for each variable in the model. *R*^2^-adjusted (adj) values given are a measure of the total variance described by the model when only the components listed are used. *R*^2^-adjusted is a modified statistical term which incorporates a correction for the positive bias of *R*^2^.

Model and Variable	*R*^2^	Coefficient	*p*-Value
**Total *Microcystis* 16S rDNA (*R*^2^-adj = 0.53, *p* < 0.001)**			
Constant	-	5.872	0.001
TP	0.359	11.441	0.000
DIC	0.480	−2.724	0.006
Temp	0.555	1.979	0.012
**Toxic *Microcystis mcyD* (*R*^2^-adj = 0.59, *p* < 0.001)**			
Constant	-	5.033	0.014
TP	0.370	10.765	0.000
Temp	0.516	2.667	0.003
DIC	0.561	−3.490	0.004
NO_3_^−^–N	0.625	3.558	0.013
**MC (*R*^2^-adj = 0.72, *p* < 0.001)**			
Constant	-	−0.162	0.835
TP	0.391	7.605	0.000
Temp	0.625	1.892	0.000
DIC	0.735	−1.816	0.000

## 3. Discussion

This investigation in Lake Chaohu explored the correlations among *Microcystis* population dynamics, MC concentrations and environmental variables. Our results suggested that toxic *Microcystis* genotypes coexisted with the non-toxic *Microcystis* genotypes, and that the abundance of *Microcystis* genotypes and MC production varied across spatial and temporal scales. These results were supported by the Pearson correlation and stepwise multiple regressions analyses that showed total phosphorus, water temperature and dissolved inorganic carbon were the primary variables regulating the variations in *Microcystis* population dynamics and MC concentrations during the bloom season.

Lake Chaohu is located in the subtropical climate zone, which the total sun radiation is about 499 kJ/(cm^2^ a), the annual mean temperature is about 16 °C, and the annual rainfall is 1100 mm. These conditions combined with the shallow water depth and high nutrient concentrations, provide the favorable conditions for cyanobacteria mass proliferation [35]. Consequently, this lake exhibits annual heavy cyanobacteria blooms, leading to higher MC concentrations. The results from the present study showed that MC concentrations were positively correlated with the abundance of *Microcystis mcyD* copies (*r* = 0.69, *p* < 0.001, *n* = 45). However, interestingly, the abundance of toxic *Microcystis* in June was relatively low at sites 7–9 in the western part of the lake (Figure 4), while MC concentrations were remarkably high and comparable with that in August, when the highest abundance of toxic *Microcystis* was observed (Figure 2, Figure 4 and Figure 6). This inconsistency may be due to the production of MCs by other species in June. In fact, the potential MC-producing cyanobacterial genera *Aphanizomenon* and *Anabaena* were observed in Lake Chaohu, especially in June when the bloom was found to be composed mainly of *Aphanizomenon*, *Anabaena* and *Microcystis*. Among these species, the average biomass of *Aphanizomenon* represented 50.6% of the total cyanobacteria, *Anabaena* comprised 28.5% and *Microcystis* accounted for 19.9% [36]. Therefore, a proportion of the MCs detected in June may have been released from *Aphanizomenon* and *Anabaena*. Although there are other potential MC-producing cyanobacteria present in Lake Chaohu, one cannot deny that *Microcystis* as the major contributor to MC production during the summer, when it was predominant in the lake.

Data from qPCR showed that in Lake Chaohu, toxic *Microcystis* genotypes coexisted with non-toxic *Microcystis* genotypes. The proportion of toxic and non-toxic *Microcystis*, which reflected the abundance of *Microcystis* 16S rDNA and *mcyD* copies, varied across sites and phases of the bloom development (Figure 2, Figure 3 and Figure 4). Proportions of toxic *Microcystis* genotypes varied from 9.43% to 87.98%, which is in agreement with previous investigations of several lakes where toxic genotypes are generally lower than the abundance of total *Microcystis* in natural algal populations [19,24,37,38]. Some of these investigations have revealed that the proportions of *mcy*-containing *Microcystis* are relatively low, such as 1%–38% for *mcyB* subpopulations in Lake Wannsee, Germany [37], 0.5%–35% for *mcyA* subpopulations in Lake Mikata, Japan [19], and 0%–37% for *mcyD* subpopulations in Lake Oneida, USA [38], while higher proportions of toxigenic *Microcystis* have been observed in Lake Volkerak, the Netherlands [13] and Lake Ronkonkoma, USA [17]. In the present study, the eastern and western regions of the lake showed low and high toxic proportions of *Microcystis*, respectively (Figure 5). This spatial difference may be explained by the fact that the eutrophication levels for the western region were significantly higher than that for eastern region. High nutrient concentrations are able to maintain high abundance of *Microcystis*, with a much higher proportion of toxigenic genotypes.

The MC concentrations showed a similar pattern as the *Microcystis* 16S rDNA and *mcyD* copies, with their highest abundance all recorded at the western site 9 in August. In accordance with the proportion of toxic *Microcystis* genotypes, the MC concentrations at the western sampling sites of Lake Chaohu were significantly higher than those at the eastern sampling sites (Figure 6). This finding and similar results from other studies [25] suggest that these changes in the MC concentrations can be attributed to variations in genotype composition within the cyanobacterial community [38]. During the study period, the MC concentrations increased as the blooms developed, and the peak value reached 17.61 μg L^−^^1^, which is similar to a previous reported by Yang *et al.* [31]. Yang and colleagues have conducted an extensive investigation of Lake Chaohu, and have found MC pollution throughout the entire lake, particularly in the western region. MCs are chemically very stable and do not readily undergo proteolytic or hydrolytic attack [39], long-term exposure to MCs in the field is expected to adversely affect the detoxification capabilities of aquatic organisms [40]. In Lake Chaohu, the tissue distribution and bioaccumulation of MCs have been reported in fish, shrimp, and mussels at different trophic levels [41,42,43], whereas fishermen frequenting this lake have hepatocellular damage and alarming serum concentrations due to chronic MC exposure [44]. Together with the current study, these data suggest that Lake Chaohu may be unsafe for fish and drinking water use and further monitoring is recommended.

*Microcystis* dynamics and MC production have been demonstrated to be associated with environmental factors in previous studies [17,45,46,47,48]. In our study, the abundance of *Microcystis* 16S rDNA and *mcyD* copies and MC concentrations showed significant positive correlations with TP, TN, NH_4_^+^–N, NO_3_^−^–N, PO_4_^3^^−^–P, water temperature, and pH. Negative correlations were observed with Secchi depth, water depth, and DIC.

According to the regression models, *Microcystis* 16S rDNA and *mcyD* copy abundances and the MC concentrations showed strong positive correlations with TP, indicating that increasing the phosphorus (P) concentration could increase the toxic *Microcystis* abundance and MC production. Our observation agreed with those from a study conducted in the large eutrophic Lake Erie in USA [24], where positive correlations between TP and the abundance of toxic *Microcystis* and MC were found. Vézie *et al.* [49] have also found that higher P concentrations are beneficial to the growth of toxic *Microcystis* and MC synthesis. Consistent with the trend, Davis *et al.* [17] have reported that the growth rate of toxic *Microcystis* exceeds the non-toxic strains under elevated P concentrations. These results could be explained by the fact that toxic *Microcystis* has significant P requirements associated with the enzymes involved in the synthesis of MC, as well as with additional light-harvesting pigments that they may possess [50,51]. Although TP was a dominant explanatory variable, the effect of nitrogen (N) on the *Microcystis* dynamics and MC production cannot be ignored. MC is an *N*-rich compound; thus, toxic *Microcystis* has additional N requirements associated with MC synthesis [50]. Previous studies have also demonstrated that increasing the N concentration could promote the growth and toxicity of *Microcystis* [52,53,54]. Furthermore, both correlation and stepwise multiple regression analyses in our study showed that toxic *Microcystis* abundance and MC concentrations were positively correlated with TN and NO_3_^−^–N, consistent with many other studies [28,31,55].

In Lake Chaohu, the higher TP and TN concentrations in the western part of the lake may have promoted favorable conditions for *Microcystis* and MC synthesis, thus leading to the increased abundance of toxic *Mirocystis* and MC production in western Lake Chaohu compared to the eastern region. Similar phenomenon was observed in Lake Taihu [56], where spatial differences in the abundance of toxic *Microcystis* and the MC concentrations were positively correlated with TP and TN. All of these results suggest that increasing nutrient concentrations, especially TP, may promote the growth of a toxic, rather than non-toxic, population of *Microcystis*, resulting in blooms with higher MC concentrations.

Temperature is the primary driving factor of the proliferation of cyanobacteria in natural freshwater [57]. Harmful cyanobacteria such as *Microcystis*, have an optimal temperature for growth at, or above, 25 °C [16,58,59]. Rising temperatures not only promote the dominance of *Microcystis* but may favour the proliferation of toxic *Microcystis* strains and result in an increase in the MC concentrations [60]. According to the models, temperature was associated with the 16S rDNA and *mcyD* copy abundances and MC concentrations, particularly in August, when the copy numbers of the 16S rDNA and *mcyD* genes at the maximum along with a peak in the total MC concentrations. This finding may support the previous hypothesis that factors increasing the growth rate of cells also enhance MC production [17,49,53,61]. Positive relationships between temperature, *Microcystis* abundance and MC production have been observed in other cyanobacterial-prone bodies of water, including Lake Taihu, in China [62] and two reservoirs, namely Hartbeespoort and Roodeplaat, in South African [25]. These results and our data support the prediction from Paerl and Huisman [16] that future global warming can lead to toxic *Microcystis* blooms of longer durations.

By contrast, the model describing the abundances of 16S rDNA and *mcyD* copies and the MC concentration showed negative correlations with DIC, suggesting that the decrease in DIC concentration was accompanied by increases in the 16S rDNA and *mcyD* copy mumbers and the MC concentrations in Lake Chaohu. Because cyanobacteria are better competitors for DIC [63,64], several taxa, including *Microcystis* are able to use bicarbonate as a carbon source when CO_2_ availability is limited [65,66]. In our study, pH was often above 8, even reaching 9 during the summer (Table 1), indicating that CO_2_ availability was quite low [67]. However, the copy numbers of 16S rDNA and *mcyD* and MC concentrations reached their peaks, supporting the hypothesis that low CO_2_ conditions, particularly in a eutrophic lake, may favour *Microcystis* growth [63,68,69]. Moreover, variations in the MC concentrations may be moderated by CO_2_ availability [70,71,72]. Additionally, Van de Waal *et al.* [73] have demonstrated that toxic *Microcystis* has a competitive advantage and grows better at low CO_2_ concentrations, thereby increasing the MC concentrations. Conversely, toxic *Microcystis* has lost this advantage and is associated with low MC concentrations under elevated CO_2_ concentrations. These results suggest that rising CO_2_ concentrations may decrease toxic *Microcystis* abundance and MC concentrations in the future.

## 4. Experimental Section

### 4.1. Sampling Sites and Collection Methods

Nine fixed sampling sites in Lake Chaohu were selected for water sampling (Figure 1). Sites 1–3 were located in the eastern part of Lake Chaohu, sites 4–6 were located in the centre of the lake, and sites 7–9 were located in the western part. Triplicate water samples were collected monthly from the surface layer (0.5 m) with a sterile sampler from June to October 2012. All samples were preserved in sterile bottles and transported to the laboratory on ice. The water samples were filtered using GF/C filters (pore size, 1.2 μm; Whatman, Maidstone, England, UK), and the filters containing the phytoplankton samples were immediately kept frozen at −80 °C until DNA extraction and MC analysis.

### 4.2. Environmental Variables

Water temperature, DO, conductivity and pH were measured *in situ* with a multiparameter water quality sonde (YSI 6600, Yellow Spring Instruments, Yellow Springs, OH, USA). Water depth was measured using a Portable Depth Sounder (SM-5, SpeedTech Instruments, Great Falls, VA, USA). Water transparency was measured with a Secchi disk. TP was analysed according to the ammonium molybdate spectrophotometric method (GB11893-89, China), and TN was determined by the alkaline potassium persulphate digestion-UV spectrophotometric method (GB11894-89, China). To determine the levels of dissolved inorganic nutrients, including PO_4_^3−^–P, NO_3_^−^–N, NO_2_^−^–N and NH_4_^+^–N, 50 mL of the water sample was filtered through a GF/F glass fiber filter (pore size, 0.8 μm; Whatman, Maidstone, England, UK) and analysed using a continuous flow analyser (San plus system, Skalar, Breda, The Netherlands) according to the manufacturer’s instructions. DIC and dissolved organic carbon (DOC) were analysed on filtrates from samples filtered through GF/F glass fiber filter (pore size, 0.8 µm; Whatman, Maidstone, England, UK; via burning in the muffle furnace for 4 h at 500 °C), and measured by high temperature burning method with a TOC analyzer (Torch, Teledyne Tekmar, Mason, OH, USA). To analyse Chl-a concentrations, the triplicate water samples were filtered through GF/C filters (pore size, 1.2 μm; Whatman, Maidstone, England, UK) and measured according to Lorenzen [74] with spectrophotometric measurements after extraction in hot 90% ethanol.

### 4.3. Microcystin (MC) Analysis

Lake water (500 mL) was filtered through a GF/C glass fiber filter (pore size, 1.2 µm; Whatman, Maidstone, England, UK) in triplicate to separate the microcystin dissolved in water (extracellular MC) and microcystin in particles (intracellular MC). For intracellular MC analysis, the filters were lyophilised and extracted with 5% (*v*/*v*) acetic acid solution followed by 80% (*v*/*v*) aqueous methanol [75], with an additional step involving the grinding of the filters using a Fast Prep-24 automated homogeniser (MP Biomedicals, Santa Ana, CA, USA) with 0.5-mm silica beads. After centrifugation (9500 r/min, 10 min), the supernatants were pooled and diluted with distilled water, and the distilled supernatants were concentrated using solid-phase extraction cartridges (C18, 0.5 g) and eluted with 100% (0.1% TFA) methanol. After being blown dry with nitrogen at 40 °C, the residues were each re-suspended in 150 µL of 50% aqueous methanol prior to HPLC analysis. Extracellular microcystin concentrations were determined by the filtration of 500 mL of lake water using GF/C filters (pore size, 1.2 µm; Whatman, Maidstone, England, UK) and were concentrated with solid phase extraction cartridges. The next steps involving nitrogen blow-drying and methanol resuspension were performed as described for the intracellular MC extraction.

MC was analysed using high-performance liquid chromatography with photodiode array detection (Agilent 1200, Agilent, Palo Alto, CA, USA) equipped with an ODS column (Agilent Eclipse XDB-C18, 5 µm, 4.6 mm × 150 mm), using a gradient of 30 to 70% (*v*/*v*) acetonitrile (with 0.05% (*v*/*v*) trifluoroacetic acid) at a flow rate of 1 mL min^−1^. MC was identified by its characteristic UV spectra. Total MC concentrations were quantified as the sum of all MC peaks using MC-LR, -RR, and -YR standards (Sigma, München, Germany).

### 4.4. DNA Extraction and Quantitative Real-Time PCR

The GF/C filters were cut into small pieces with a sterile scalpel. Total DNA was extracted using the potassium xanthogenate sodium dodecyl sulfate method as described previously [76].

Real-time PCR assay was used to quantify the 16S rDNA and *mcyD* gene regions. The 16S rDNA gene primers 184F (5'-GCCGCRAGGTGAAAMCTAA-3') and 431R (5'-AATCCAAARACCTTCCTCCC-3') were used to quantify the abundance of the total *Microcystis* population [77], whereas the *mcyD* gene primers F2 (5'-GGTTCGCCTGGTCAAAGTAA-3') and R2 (5'-CCTCGCTAAAGAAGGGTTGA-3') were used to detect the abundance of the toxic *Microcystis* population [78].

To construct a standard curve for real-time PCR analysis, MC-producing *Microcystis aeruginosa* PCC 7806, which was obtained from the FACHB-Collection (Freshwater Algal Culture Collection of the Institute of Hydrobiology, China), was used as a standard strain. The standard curves showing the relationships between the target gene copy numbers and the threshold cycles (*C_t_*) were generated with serial dilutions of genomic DNA. *M. aeruginosa* 7806 was grown in BG-11 medium at 25 °C and 60 μmol photons m^−2^·s^−1^. Cells from a known volume of the *M. aeruginosa* PCC 7806 culture were filtered through GF/C filters, and the DNA extraction was conducted as described above. The DNA concentration (ABS260) and purity (ABS260/ABS280) were determined spectrophotometrically. A 10-fold dilution series of the DNA was prepared, and the 16S rDNA and *mcyD* genes were amplified by real-time PCR. The copy numbers of these two genes above were calculated according to Vaitomaa *et al.* [21].

Real-time PCR was performed with a *Mastercycler realplex* 4 system (Eppendorf, Hamburg, Germany) using 25 µL of a reaction mixture, which containing 12.5 µL of SYBR Premix EX Taq™ (TaKaRa, Kusatsu, Japan), 10 µM of each primer, 10.5 µL of distilled water, and 1 µL of the template DNA. All of the samples were amplified in triplicate. The negative controls without the template DNA were included for each PCR run. Reaction conditions for amplifying 16S rDNA and *mcyD* were as follows: 95 °C for 2 min, followed by 40 cycles of 95 °C for 30 s, 55 °C for 30 s, and 72 °C for 30 s. The melting temperature for the real-time PCR products was determined using the manufacturer’s software.

### 4.5. Statistical Analysis

The relationships between the *Microcystis* genotypes and MC production as well as the environmental factors were assessed by Pearson correlations. To obtain the significant factors that explained the occurrences of the *Microcystis* genotypes and MC production, stepwise multiple regressions were conducted. All of the environmental variables, including TP, TN, NH_4_^+^–N, NO_3_^−^–N, NO_2_^−^–N, PO_4_^3^^−^–P, DOC, DIC, temperature, pH, DO, conductivity, water depth and Secchi depth, were included in the stepwise multiple linear regression analyses. Environmental variables, except pH, were log_10_ (*x* + 1) transformed before analysis to meet the conditions of normality and homogeneity of variance in the residuals. The variance inflation factor (VIF) was used to test the colinearity among variables, and variables with a VIF value of greater than 10 were considered to be autocorrelated with the other(s). The R^2^ value was used to identify the best model, and variables within the stepwise multiple regressions model were deemed significant at a P-value of less than 0.05. All of the statistical analyses were conducted using SPSS16.0 software for Windows (SPSS Inc., Chicago, IL, USA). Additionally, the Kruskal–Wallis nonparametric test was used to determine the differences between the different sampling sites during the bloom in the lake.

## 5. Conclusions

Environmental variables that affect *Microcystis* population dynamics and MC production were examined in the large, shallow, eutrophic Lake Chaohu. The results of our study demonstrated that total phosphorus, water temperature, and dissolved inorganic carbon were key variables influencing *Microcystis* dynamics and MC distribution across spatial and temporal scales. It is clear that eutrophication coupled with global warming can lead to more frequent toxic blooms and greater concentrations of MC. However, rising CO_2_ levels may suppress toxic *Microcystis* abundance and MC concentrations in the future. Considering the full complexity of aquatic ecosystems, changes in multiple environmental variables will not occur independent of each other, but rather simultaneously. This highlights the need for further field research to obtain a better understanding of the synergistic effects of temperature, CO_2_ and nutrients on the severity and toxicity of *Microcystis* blooms. Such information is important for monitoring and predicting the potential health risks associated with toxic *Microcystis* blooms, which fluctuate in response to both eutrophication and climate change. Moreover, effective management strategies can be implemented to prevent the further deterioration of the water quality in Lake Chaohu and elsewhere.

## Supplementary Materials

Supplementary materials can be accessed at: http://www.mdpi.com/2072-6651/6/12/3238/s1.
